# Anemia is a risk factor for rapid eGFR decline in type 2 diabetes

**DOI:** 10.3389/fendo.2023.1052227

**Published:** 2023-01-23

**Authors:** Lijie Xie, Xiaoqing Shao, Yifei Yu, Wei Gong, Fei Sun, Meng Wang, Yeping Yang, Wenjuan Liu, Xinmei Huang, Xia Wu, Huihui Wu, Yiming Li, Zhaoyun Zhang, Jie Wen, Min He

**Affiliations:** ^1^ Department of Endocrinology and Metabolism, Huashan Hospital, Fudan University, Shanghai, China; ^2^ Department of Endocrinology and Metabolism, Jingan Branch of Huashan Hospital, Shanghai, China

**Keywords:** glomerular filtration rate, anemia, type 2 diabetes mellitus, diabetic kidney disease, rapid eGFR decline

## Abstract

**Objective:**

To investigate the association between anemia and progression of diabetic kidney disease (DKD) in type 2 diabetes.

**Methods:**

This was a retrospective study. A total of 2570 in-patients with type 2 diabetes hospitalized in Jinan branch of Huashan hospital from January 2013 to October 2017 were included, among whom 526 patients were hospitalized ≥ 2 times with a median follow-up period of 2.75 years. Annual rate of eGFR decline was calculated in patients with multiple admissions. A rate of eGFR decline exceeding -5 ml/min per 1.73 m^2^ per year was defined as rapid eGFR decline. The prevalence of DKD and clinical characteristics were compared between anemia and non-anemia patients. Correlation analysis was conducted between anemia and clinical parameters. Comparison of clinical features were carried out between rapid eGFR decline and slow eGFR decline groups. The risk factors for rapid DKD progression were analyzed using logistic regression analysis.

**Results:**

The prevalence of anemia was 28.2% among the 2570 diabetic patients, while in patients with DKD, the incidence of anemia was 37.8%. Patients with anemia had greater prevalence of DKD, higher levels of urinary albumin-to-creatinine ratio (UACR), serum creatinine, BUN, urine α1-MG, urine β2-MG, urine NAG/Cr, hsCRP, Cystatin C, homocysteine and lower eGFR, as compared to the patients without anemia. Anemia was correlated with age, UACR, eGFR, urinary NAG/Cr, hsCRP and diabetic retinopathy (DR). Logistic regression analysis of 526 patients with type 2 diabetes during the follow-up period showed that anemia was an independent risk factor for rapid eGFR decline.

**Conclusion:**

Anemia is associated with worse renal function and is an independent risk factor for rapid eGFR decline in type 2 diabetes.

## 1 Introduction

Type 2 diabetes mellitus (T2DM) is one of the most important chronic metabolic diseases with a prevalence of 12.8% in China (1). Being one major complication of diabetes, diabetic kidney disease (DKD) has become the leading cause of end-stage renal disease worldwide (2–4). However, factors for prediction of DKD progression are not fully understood currently.

Anemia is common in patients with diabetes (5). The prevalence of anemia in patients with diabetes, including type 2 and type 1 diabetes, was reported from 14% to 45%, which was much higher than that in the general population (6, 7). In patients with DKD, a greater risk of anemia was observed than those without DKD, and anemia was usually considered as a consequence of DKD (8). In patients with DKD, the mechanisms involved in the occurrence of anemia include inappropriate response to erythropoietin (EPO), glomerular hyperfiltration, autonomic neuropathy, proteinuria, chronic inflammation, tubular dysfunction, renal interstitial fibrosis and activated renin angiotensin system (RAS) (9). However, accumulating evidences suggest that anemia is not only the result of kidney impairment, but may be involved in the pathogenesis of kidney damage in diabetes. In RENAAL study, hemoglobin level was reported to be an independent risk factor for nephropathy progression in diabetes (10). Another study showed that the severity of renal damage was particularly strongly associated with baseline low hemoglobin levels in patients with type 2 diabetes (11). However, Gu et al. found that anemia had no significance of predicting the progression of DKD after glomerular filtration rate (GFR) was adjusted (12). Therefore, the relationship between anemia and DKD remains unclear and controversial currently. And the value of anemia in predicting DKD progression needs further investigation.

To examine the association between DKD and anemia, we investigated the characteristics of anemia in patients with type 2 diabetes and explored the association of anemia with DKD.

## 2 Materials and methods

### 2.1 Patients

This was a retrospective study. 2570 patients with type 2 diabetes hospitalized in Jinan branch of Huashan hospital, from January 2013 to October 2017 were included, among whom 526 patients were hospitalized ≥ 2 times during a median follow-up period of 2.75 years. Type 2 diabetes was identified according to the American Diabetes Association criteria. DKD was diagnosed based on the 2020 guideline of KDIGO (13), which was characterized by increased urinary albuminuria level (urinary albumin creatinine ratio ≥ 30mg/g) and/or eGFR<60 ml/min/(1.73 m^2^), while excluding CKD induced by other causes. Rapid eGFR decline was defined by a rate of eGFR decline exceeding -5 ml/min per 1.73 m^2^ per year (14). The diagnostic criteria for anemia were Hb < 120 g/L in females and Hb < 130 g/L in males (15). This study was approved by the ethics committee of Huashan Hospital (No. HIRB-395). Written informed consent was obtained from all patients.

### 2.2 Clinical data and laboratory examination

The clinical data were collected according to medical records. Clinical data included age, gender, clinical manifestations. Laboratory examinations of blood include hemoglobin (Hb), mean cell volume (MCV), mean cell hemoglobin concentration (MCHC), serum creatinine (sCr), blood urea nitrogen (BUN), blood uric acid (UA), triglyceride (TG), total cholesterol (TC), low density lipoprotein cholesterol (LDL-C), high density lipoprotein cholesterol (HDL-C), apolipoprotein A (apoA), apolipoprotein B (apoB), lipoprotein a [Lp(a)], hypersensitive C reactive protein (hs-CRP), homocysteine, fast blood glucose (FBG), glycosylated hemoglobin (HbA1c) and glycated albumin. Urine tests included 24-hour urine albumin, urine Albumin-Creatinine-Ratio (UACR), urine N-Acetyl-β-D-glucosaminidase (NAG), urine α-Microglobulin (α1MG). EGFR was calculated using the modified MDRD formula for the Chinese population as eGFR (ml/min per 1.73 m^2^) = 175 × sCr^-1.234^ × age^-0.179^ (if female × 0.79) (16).

### 2.3 Statistical analysis

Statistical analysis was carried out using SPSS software (version 20.0, Chicago, IL, USA). Data of normal distribution was shown as mean ± standard deviation (m ± SD) and compared using unpaired *t* test. Data of non-normal distribution were described as median (interquartile range) and analyzed using non-parametric tests. Qualitative data were expressed in frequency and analyzed using χ^2^ test. Logistic regression was performed to analyze the relationship between anemia and rapid eGFR decline. P value less than 0.05 was regarded as statistically significant.

## 3 Results

### 3.1 Clinical characteristics of patients

A total of 2570 patients with type 2 diabetes were included in this study, including 1449 males (56.4%) and 1121 females (43.6%). Of all the patients, 966 patients (male 525 and female 441) were diagnosed as DKD with a prevalence of 37.59% (36.23% in males and 39.34% in females, respectively) at baseline (**Table 1**). The age of patients with DKD was older than those without DKD (68 [60-79] vs. 64 [57-72] years, p < 0.0001). In addition, higher levels of hsCRP (3.9 [2.2-9.0] vs. 2.5 [1.7-4.7] mg/L, p < 0.0001), TG (1.56 [1.07-2.29] vs. 1.36 [0.96-2.00] mmol/L, p < 0.0001), apoB (0.86 [0.72-1.03] vs. 0.84 [0.70-0.98] g/L, p < 0.0001), Lp(a) (18 [8-122.75] vs. 14 [8-108] mg/L, p = 0.001), FBG (8.46 [6.39-12.37] vs. 7.52 [5.9-10.1] mmol/L, p < 0.0001), HbA1c (8.9% vs. 8.3%, p < 0.0001) and glycated albumin (23.4% vs. 21.7%, p < 0.0001), and lower concentrations of HDL-C (0.92 [0.78-1.08] vs. 0.98 [0.84-1.17] mmol/L, p < 0.0001) and apoA (1.08 [0.93-1.29] vs. 1.14 [0.97-1.38] g/L, p < 0.0001) were observed in the patients of DKD, as compared to the patients without DKD (**Table 1**).

**Table 1:** Clinical characteristics of type 2 diabetic patients with or without DKD.

Variable	Without-DKD	DKD	p value
**No.**	1604	966	–
**Gender (M/F)**	924/680	525/441	0.107
**Age (years)**	64 (57-72)	68 (60-79)	< 0.0001^****^
**Anemia (%)**	22.44	37.78	< 0.0001^****^
**Hb (g/L)**	135 (125-145)	129 (117-141)	< 0.0001^****^
**MCV (fl)**	90 (88-93)	89 (87-93)	< 0.0001^****^
**MCHC (g/L)**	333 (325-342)	334 (325-343)	0.237
**UACR (mg/g)**	9.11 (5.92-14.68)	110.17 (48.45-361.54)	< 0.0001^****^
**sCr (μmol/L)**	64 (53-74)	77 (60-106)	< 0.0001^****^
**BUN (mmol/L)**	4.59 (3.82-5.63)	5.59 (4.36-7.43)	< 0.0001^****^
**Uric acid (μmol/L)**	279 (233-332)	309 (254-375.25)	< 0.0001^****^
**Cystatin C (mg/g)**	0.86 (0.72-1.02)	1.07 (0.85-1.4)	< 0.0001^****^
**eGFR (ml/min per1.73 m^2^)**	105 (89-124)	84 (55-109)	< 0.0001^****^
**Urine β2MG (mg/L)**	1.89 (1.58-2.33)	2.43 (1.89-3.38)	< 0.0001^****^
**Homocysteine (umol/L)**	12.2 (10.4-14.4)	13.3 (11.2-15.8)	< 0.0001^****^
**Urine albumin (mg/24h)**	8 (4-13)	77 (30-243)	< 0.0001^****^
**Urine NAG (U/L)**	15 (10-24)	19 (12-29)	< 0.0001^****^
**Urine NAG/Cr (U/mmol)**	2.13 (1.46-3.27)	3.35 (2.27-5.17)	< 0.0001^****^
**Urine α1MG (mg/L)**	9.29 (5.27-16.1)	19 (9.92-37.6)	< 0.0001^****^
**α1MG/Cr (mg/g)**	1.49 (0.99-2.42)	3.61 (1.93-7.11)	< 0.0001^****^
**hsCRP (mg/L)**	2.5 (1.7-4.7)	3.9 (2.2-9.0)	< 0.0001^****^
**Cholesterol (mmol/L)**	4.42 (3.75-5.17)	4.44 (3.73-5.24)	0.605
**Triglyceride (mmol/L)**	1.36 (0.96-2.00)	1.56 (1.07-2.29)	< 0.0001^****^
**LDL-C (mmol/L)**	2.57 (2.03-2.57)	2.56 (1.96-3.26)	0.736
**HDL-C (mmol/L)**	0.98 (0.84-1.17)	0.92 (0.78-1.08)	< 0.0001^****^
**apoA (g/L)**	1.14 (0.97-1.38)	1.08 (0.93-1.29)	< 0.0001^****^
**apoB (g/L)**	0.84 (0.70-0.98)	0.86 (0.72-1.03)	< 0.0001^****^
**Lpa (mg/L)**	14 (8-108)	18 (8-122.75)	0.001^**^
**HbA1c%**	8.3 (7-10.1)	8.9 (7.5-10.7)	< 0.0001^****^
**FBG (mmol/L)**	7.52 (5.9-10.1)	8.46 (6.39-12.37)	< 0.0001^****^
**Glycated albumin (%)**	21.7 (17.3-27.9)	23.4 (18-29.8)	< 0.0001^****^

Data were shown as median (interquartile range).

**p < 0.01; ****p < 0.0001.

### 3.2 Prevalence of anemia in hospitalized type 2 diabetic patients

Of the 2570 diabetic patients, there were 725 patients (male 367 and female 358) having anemia, with a prevalence of 28.2% (25.3% in males and 31.9% in females) (**Table 2**). Among 1604 patients without DKD, 360 patients (male 177 and female 183) were diagnosed having anemia, with a prevalence of 22.44%. In 966 patients with DKD, 365 patients (male 190 and female 175) were diagnosed having anemia, with a prevalence of 37.78%, which was much higher than that in patients without DKD (p < 0.0001) (**Table 1**).

**Table 2:** Clinical characteristics of type 2 diabetic patients with or without anemia.

Variable	No-anemia	anemia	p value
**No.**	1845	725	–
**Gender (M/F)**	1082/763	367/358	< 0.0001^****^
**Age (years)**	63 (56-71)	72 (63-80)	< 0.0001^****^
**DKD (%)**	32.57	50.34	< 0.0001^****^
**Hb (g/L)**	139 (131-147)	115 (107-120)	< 0.0001^****^
**MCV (fl)**	90 (87-93)	90 (87-94)	0.098
**MCHC (g/L)**	335 (327-344)	330 (321-339)	< 0.0001^****^
**Urinary ACR (mg/g)**	14.54 (7.16-51.39)	29.22 (10.52-174.18)	< 0.0001^****^
**sCr (μmol/L)**	65 (54-78)	74 (59-97)	< 0.0001^****^
**BUN (mmol/L)**	4.76 (3.89-5.80)	5.39 (4.21-7.21)	< 0.0001^****^
**Uric acid (μmol/L)**	293 (243-351)	283.5 (228.25-346.75	0.012^*^
**Cystatin C (mg/g)**	0.88 (0.73-1.06)	1.05 (0.85-1.34)	< 0.0001^****^
**eGFR (ml/min per1.73 m2)**	104 (85-123)	85 (60-107)	< 0.0001^****^
**Urine β2MG (mg/L)**	1.93 (1.60-2.40)	2.51 (1.95-3.51)	< 0.0001^****^
**Homocysteine (μmol/L)**	12.4 (10.5-14.6)	13.1 (11-15.9)	< 0.0001^****^
**Urine albumin (mg/24h)**	13 (6-41)	17 (7-93.75)	< 0.0001^****^
**Urine NAG (U/L)**	16 (10-26)	16 (11-26.25)	0.867
**Urine NAG/Cr (U/mmol)**	2.33 (1.55-3.66)	3.18 (2.00-5.17)	< 0.0001^****^
**Urine α1MG (mg/L)**	11 (5.93-20)	15.9 (7.53-33.95)	< 0.0001^****^
**α1MG/Cr (mg/g)**	1.68 (1.08-3.00)	3.35 (1.70-7.40)	< 0.0001^****^
**hsCRP (mg/L)**	2.8 (1.8-5.0)	3.75 (1.90-9.70)	< 0.0001^****^
**Cholesterol (mmol/L)**	4.58 (3.88-5.33)	4.1 (3.46-4.82)	< 0.0001^****^
**Triglyceride (mmol/L)**	1.51 (1.06-2.22)	1.2 (0.85-1.77)	< 0.0001^****^
**LDL-C (mmol/L)**	2.66 (2.09-3.28)	2.33 (1.82-2.93)	< 0.0001^****^
**HDL-C (mmol/L)**	0.96 (0.83-1.14)	0.94 (0.80-1.13)	0.049^*^
**apoA (g/L)**	1.12 (0.96-1.36)	1.10 (0.94-1.29)	0.002^**^
**apoB (g/L)**	0.87 (0.73-1.02)	0.79 (0.65-0.93)	< 0.0001^****^
**Lpa (mg/L)**	15 (8-118)	17 (8-106)	0.243
**HbA1c (%)**	8.7 (7.35-10.5)	7.9 (6.7-9.8)	< 0.0001^****^
**FBG (mmol/L)**	7.98 (6.21-10.86)	7.39 (5.72-10.96)	0.006^**^
**Glycated albumin (%)**	22.4 (17.7-28.6)	21.9 (17.3-28.5)	0.690

Data were shown as median (interquartile range).

*p < 0.05; **p < 0.01; ****p < 0.0001.

### 3.3 The correlation of anemia and clinical parameters in diabetic patients

Compared with the patients without anemia, patients with anemia had significantly higher levels of UACR (14.54 [7.16-51.39] vs. 29.22 [10.52-174.18] mg/g, p < 0.0001), sCr (65 [54-78] vs. 74 [59-97] μmol/L, p < 0.0001), BUN (4.76 [3.89-5.80] vs. 5.39 [4.21-7.21] mmol/L, p < 0.0001), Cystatin C (0.88 [0.73-1.06] vs. 1.05 [0.85-1.34] mg/g, p = 0.012), homocysteine (12.4 [10.5-14.6] vs. 13.1 [11.0-15.9] μmol/L, p < 0.0001), urine α1 MG/Cr (1.68 [1.08-3.00] vs. 3.35 [1.70-7.40] mg/g, p < 0.0001), urine β2 MG (1.93 [1.60-2.40] vs. 2.51 [1.95-3.51] mg/L, p < 0.0001), urine NAG/Cr (2.33 [1.55-3.66] vs. 3.18 [2.00-5.17] U/mmol, p < 0.0001), hsCRP (2.8 [1.8-5.0] vs. 3.75 [1.9-9.7] mg/L, p < 0.0001), while lower eGFR (104 [85-123] vs. 85 [60-107] ml/min per 1.73 m^2^, p < 0.0001) (**Table 2**). Logistic analysis was conducted to explore the correlations of anemia and clinical parameters in the patients of diabetes. We found that lower eGFR, females, older age, higher UACR, higher NAG/Cr, higher α1MG/Cr, higher hsCRP, diabetic retinopathy history were associated with increased prevalence of anemia among patients with T2DM (**Table 3**).

**Table 3:** Logistic analysis of anemia and clinical characteristics of diabetic patients.

Variable	B	SE	Wald	Sig.	Exp (B)	95% CI
**Female**	0.052	0.113	0.212	0.645	1.053	0.844-1.314
**Age (years)**	0.038	0.005	49.318	< 0.0001^****^	1.039	1.028-1.050
**UACR (mg/g)**	0.0003	0.000	9.503	0.002^**^	1.000	1.000-1.000
**eGFR (ml/min per1.73 m^2^)**	-0.007	0.002	12.161	< 0.0001^****^	0.993	0.989-0.997
**NAG/Cr (U/mmol)**	0.096	0.027	12.803	< 0.0001^****^	1.101	1.045-1.161
**α1MG/Cr (mg/g)**	0.023	0.012	3.763	0.052	1.023	1.000-1.048
**hsCRP (mg/L)**	0.007	0.002	9.489	0.002^**^	1.007	1.003-1.012
**DR**	0.326	0.128	6.518	0.011^*^	1.385	1.079-1.779
**Constant**	-3.524	0.477	54.662	0.000	0.029	

*p < 0.05; **p < 0.01; ****p < 0.0001.

### 3.4 The eGFR and UACR stages in diabetic patients with different Hb concentrations

**Figure 1:**
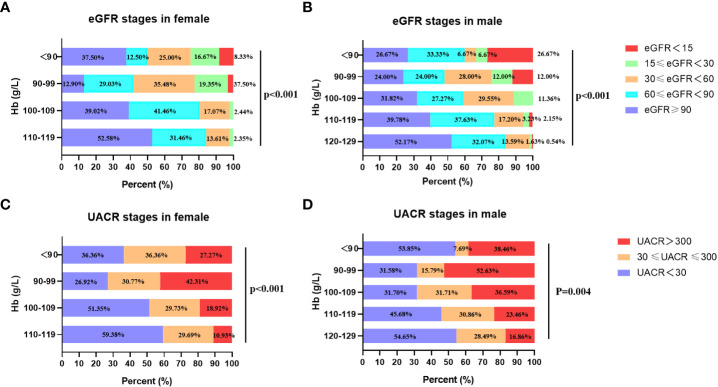
The eGFR and UACR stages in different Hb levels in females and males. **(A)** The relationship between eGFR stages and stratified Hb concentrations in female. **(B)** The relationship between eGFR stages and stratified Hb concentrations in male. **(C)** The relationship between UACR stages and stratified Hb concentrations in female. **(D)** The relationship between UACR stages and stratified Hb concentrations in male.

To investigate the relationship of renal function with anemia, the UACR and eGFR stages were analyzed in patients with different Hb levels. The eGFR were divided into 3 stages: eGFR ≥ 30 ml/min per 1.73m^2^, and eGFR < 30 ml/min per 1.73m^2^. The UACR were divided into 2 groups: UACR < 300 mg/g and UACR ≥ 300mg/g. The patients were divided into 5 groups according to baseline Hb level: 120-129 g/L, 110-119 g/L, 100-109 g/L, 90-99 g/L, Hb < 90 g/L. Given the gender difference in Hb level, gender-based subgroup analysis was performed. In male group, the percentages of eGFR < 30 ml/min per 1.73m^2^ (eGFR stages 4 and 5) in different Hb stratification (120-129 g/L, 110-119 g/L, 100-109 g/L, 90-99 g/L, Hb < 90 g/L) were 2.17%, 5.38%,11.36%, 24.00%, 33.34% respectively (**Figure 1B**), which showed that with the aggravation of anemia, the proportion of lower eGFR gradually increased. The result was similar in the female group (**Figure 1A**). In addition, the prevalence of macroalbuminuria in different Hb stratification (120-129 g/L, 110-119 g/L, 100-109 g/L, 90-99 g/L, Hb < 90 g/L) were 16.86%, 23.46%, 36.59%, 52.63% and 38.46%, respectively (**Figure 1D**) in male group. Accordantly, patients with macroalbuminuria increased as Hb level decreased in female group. In patients whose Hb≥90g/L, the proportion of macroalbuminuria and microalbuminuria increased with the aggravation of anemia in both genders (**Figures 1C, D**). But the trend was not observed in patients whose Hb < 90g/L. This may due to that patients with relatively severe anemia received more treatment. These results suggested that anemia was associated with worse renal function as shown in **Figure 1**.

### 3.5 Risk factors for rapid eGFR decline

Of the 2570 patients, 526 patients (male 299 and female 227) had been hospitalized ≥ 2 times. The interval between the first and the last hospitalized time was 18 (9.5, 32) months. Of the 526 patients, 268 (male 151 and female 117) had rapid eGFR decline (**Table 4**). The annual eGFR decline in the rapid progression group was significantly higher than that in the slow progression group (-15.49 [-26.63-(-9.74)] vs. 2.07 [-1.57-(10.96)] ml/min per 1.73m^2^ per year, p < 0.0001). To examine the risk factors for rapid eGFR decline, the baseline clinical characteristics were analyzed (**Table 4**). The rapid eGFR decline group had higher baseline eGFR (104 [82.25-123.75] vs. 88 [67-104.5] ml/min per 1.73m^2^ per year, p < 0.0001), UACR (31.65 [10.52-249.14] vs. 20.87 [9.37-78.51] mg/g, p = 0.009), urine NAG/Cr (2.78 [1.8-4.07] vs. 2.36 [1.54-3.34] U/mmol, p = 0.009), HbA1c (8.9 vs. 7.9%, p < 0.0001) and lower serum creatinine (65 [53-80] vs. 75 [62-91] μmol/L, p < 0.0001) as compared to slow group (**Table 4**). eGFR was divided into 5 stages: stage 1 means eGFR ≥ 90 ml/min per 1.73m^2^, stage 2 means 60 ≤ eGFR < 90 ml/min per 1.73m^2^, stage 3 means 30 ≤ eGFR < 60 ml/min per 1.73m^2^, stage 4 means 15 ≤ eGFR < 30 ml/min per 1.73m^2^, and stage 5 means eGFR < 15 ml/min per 1.73m^2^. The proportion of different eGFR stages was compared between the rapid decline and slow decline groups. In the rapid decline group, the proportion of stage 1 was much higher than that in slow decline group (87.5% vs. 40.5%, p<0.0001) (**Table 5**). Relatively lower Hb level and higher anemia prevalence were observed in rapid group although there was no statistical significance. Logistic analysis was performed to analyze the risk factors for rapid eGFR decline. After adjusting for baseline gender, age, UACR, serum creatinine, homocysteine, urine NAG/Cr and HbA1c, which were reported as risk factors of eGFR decline in previous studies (17–20), our logistic regression analysis showed there was a significant correlation between anemia and rapid eGFR decline, which indicated that anemia was an independent risk factor of rapid eGFR decline. Anemia increased the risk of rapid eGFR decline by 66% (**Table 6**).

**Table 4:** Clinical characteristics of type 2 diabetic patients with rapid or slow eGFR decline during follow-up.

Variable	Rapid eGFR decline	Slow eGFR decline	p value
**No.**	268	258	–
**Gender (M/F)**	151/117	148/110	0.813
**Age (years)**	65.5 (59-76)	68 (59-77)	0.148
**Hb (g/L)**	130 (121-139)	132.5 (121-144)	0.156
**Anemia (%)**	31.3	28.3	0.445
**Urinary ACR (mg/g)**	31.65 (10.52-249.14)	20.87 (9.37-78.51)	0.009^**^
**SCr (μmol/L)**	65 (53-80)	75 (62-91)	< 0.0001^****^
**BUN (mmol/L)**	5.03 (4.03-6.09)	5.08 (4.11-6.7)	0.310
**Uric acid (μmol/L)**	302 (237-357)	301 (246.5-357.5)	0.526
**Cystatin C (mg/g)**	0.96 (0.81-1.17)	1.00 (0.81-1.25)	0.319
**eGFR (ml/min per1.73 m^2^)**	104 (82.25-123.75)	88 (67-104.5)	< 0.0001^****^
**eGFR decline per year (ml/min per 1.73m^2^ per year)**	-15.49 (-26.63-(-9.74))	2.07 (-1.57-10.96)	< 0.0001^****^
**Homocysteine (μmol/L)**	13 (11.1-15.1)	13.2 (11.3-15.7)	0.081
**Urine albumin (mg/24h)**	23.5 (8-154.5)	17 (6-56)	0.009^**^
**Urine NAG (U/L)**	16 (10-25)	13.5 (9-25)	0.153
**Urine NAG/Cr (U/mmol)**	2.78 (1.8-4.07)	2.36 (1.54-3.34)	0.009^**^
**Urine α1MG (mg/L)**	12.9 (6.27-23.8)	13.25 (6.44-23.7)	0.984
**α1MG/Cr (mg/g)**	2.17 (1.29-4.53)	2.17 (1.21-4.19)	0.644
**hsCRP (mg/L)**	3.1 (1.8-5.6)	2.8 (1.6-5.4)	0.514
**Cholesterol (mmol/L)**	4.49 (3.73-5.14)	4.36 (3.71-5.15)	0.642
**Triglyceride (mmol/L)**	1.45 (1.03-2.17)	1.51 (1.04-2.06)	0.822
**LDL-C (mmol/L)**	2.57 (1.90-3.30)	2.57 (1.95-3.23)	0.890
**HDL-C (mmol/L)**	0.93 (0.79-1.11)	0.94 (0.83-1.12)	0.377
**apoA (g/L)**	1.14 (0.97-1.38)	1.16 (1.01-1.37)	0.869
**apoB (g/L)**	0.83 (0.71-0.97)	0.82 (0.67-0.97)	0.433
**Lpa (mg/L)**	10 (8-34)	9 (8-22)	0.163
**HbA1c (%)**	8.9 (7.35-10.60)	7.9 (6.75-9.25)	< 0.0001^****^
**FBG (mmol/L)**	8.27 (6.15-11.88)	7.57 (5.99-11.18)	0.347
**Glycated albumin (%)**	23.6 (17.85-29.75)	19.65 (15.98-24.93)	< 0.0001^****^

Data were shown as median (interquartile range).

**p < 0.01; ****p < 0.0001.

**Table 5:** Proportion of different eGFR stages in rapid eGFR decline and slow eGFR decline groups.

Variable	eGFR stages					P value
	1	2	3	4	5	
**Slow eGFR decline**	143(40.5%)	135(38.2%)	64(18.1%)	9(2.6%)	2(0.6%)	< 0.0001^****^
**Rapid eGFR decline**	168(87.5%)	21(10.9%)	2(1.1%)	0(0.0%)	1(0.5%)	

The eGFR were divided into 5 stages: stage 1: eGFR ≥ 90 ml/min per 1.73m^2^, stage 2: 60 ≤ eGFR < 90 ml/min per 1.73m^2^, stage 3: 30 ≤ eGFR < 60 ml/min per 1.73m^2^, stage 4: 15 ≤ eGFR < 30 ml/min per 1.73m^2^, and stage 5: eGFR < 15 ml/min per 1.73m^2^.

**Table 6:** Relationship between anemia and rapid eGFR decline.

Models	OR	95% CI	p value
Model 1	1.16	0.80-1.68	0.445
Model 2	1.25	0.85-1.83	0.257
Model 3	1.35	0.91-2.02	0.140
Model 4	1.66	1.02-2.69	0.041^*^

Model 1: univariable.

Model 2: adjusted for gender and age.

Model 3: adjusted for gender, age and HbA1c%.

Model 4: adjusted for gender, age, HbA1c%, serum creatinine, UACR, urine NAG/Cr, homocysteine

*p < 0.05.

## 4 Discussion

Anemia is common in patients with type 2 diabetes (5). The incidence of anemia in diabetic patients were reported as 14%-45%. In this study, the prevalence and characteristics of anemia were analyzed in 2570 Chinese hospitalized type 2 diabetic patients. The incidence of anemia in our study was 28.2%, which was consistent with previous reports. Altogether, studies from our group and others suggested that patients with type 2 diabetes had higher risk of anemia.

In the early opinion, the high prevalence of anemia in diabetic patients was caused by kidney damage (21, 22). As shown in this study, the prevalence of anemia was up to 37.8% in patients with DKD, which was higher than that in patients without DKD. Compared with diabetic patients without anemia, worse renal function was shown in the patients with anemia. In addition, lower eGFR and higher UACR were correlated with lower Hb levels. These results suggested anemia was a consequence of renal damage. However, an increasing prevalence of anemia was also observed in diabetic patients with normal renal function (23). Among our patients, the prevalence of anemia was also increased in patients without DKD compared with general population. Up to 22.44% of diabetic patients without DKD had anemia in our cohort. The results suggested that anemia may not only be the consequence of renal damage in diabetic patients, but also a contributor to the progression of DKD. Anemia may occur before the development of DKD (24). Other causes were also involved in the occurrence of anemia in diabetic patients. Renal damage, however, aggravated anemia in type 2 diabetic patients. Our study found that eGFR rapid decline group had a better baseline renal function than slow decline group. Interestingly, Krolewski et al. also observed this paradoxical phenomenon in Joslin T1DM ESRD Cohort. They analyzed the clinical characteristics at entry in their cohort and found that median eGFR at entry was higher in the fast eGFR decliners. They speculated that most likely, the slow decliners’ low eGFR at entry reflected that the onset of their slow decline began many years before entry into the study and was not captured in their data (25). This may also be the explanation of our results.

The mechanisms underlying the high incidence of anemia in diabetic patients are not fully understood. Deficiency of iron and erythropoietin (EPO), insensitive response to EPO are the potential pathogenesis. Abnormal diet or iron absorption may lead to iron deficiency in diabetic patients. The production of EPO is regulated by the sympathetic nervous system. Renal sympathetic dysfunction in diabetic patients may induce renal interstitial hypoxia and EPO deficiency (26, 27). Renal tubular cell apoptosis and renal tubular ischemia induced by chronic hyperglycemia, hypoxia induced factor 1 (HIF-1) and renal tubulointerstitial fibrosis also disturbs the EPO synthesis (28, 29). In addition, the increased circulating inflammatory cytokines, such as TNF-α, IL-6, TGF-β, augment the apoptosis of erythroid progenitor cells. Oxidative stress aggravated by chronic hyperglycemia and advanced glycation end products (AGEs) also decreases deformability in erythrocytes and shortened their life expectancy (30). Furthermore, hypoglycemic agents such as metformin, thiazolidinediones (TZD), dipeptidyl peptidase-4 (DPP-4) inhibitor were also confirmed to be associated with anemia (31–35). Among the diabetic patients enrolled in our study, higher hsCRP levels was observed in patients with anemia than those without anemia. This suggested more severe inflammation existed in anemic patients. Meanwhile, patients with anemia had higher urinary β2-MG and NAG/Cr, suggesting more aggravated injury of renal tubules. Inflammation and renal tubular injury may be involved in the pathogenesis of anemia. The role of EPO, iron metabolism and medicine in development of anemia could not be determined in our cohort since the absence of these information.

Anemia is not only a symptom of kidney injury, but may aggravate kidney damage. Hypoxia resulted from anemia regulates a series of genes involved in angiogenesis, vasomotion, glycolysis, matrix metabolism, and cell survival, which may augment mitosis and fibrosis in the kidney. In addition, red blood cells are the main antioxidants in the circulation. The level of hemoglobin is closely related to oxidative stress and may be involved in the pathogenesis of DKD (36). Our study showed anemia was associated with lower eGFR, massive albuminuria and aggravated tubular injury in type 2 diabetic patients.

Since anemia may occur before the development of DKD, and lower Hb levels were associated with lower eGFR and higher levels of renal tubular injury markers, could anemia be a predictor for the progression of DKD? In order to answer this question, anemia and other risk factors for rapid eGFR decline were analyzed in this study. There was a significant correlation between anemia and rapid eGFR decline in model 4, but there was no statistical difference in model 1 to model 3, which indicated that the confounding factors including serum creatinine, UACR, urine NAG/Cr, and homocysteine may have an impact on the relationship of anemia and rapid eGFR decline. After adjusting these confounding factors, statistical differences could be obtained, which indicated that the effect of anemia on eGFR was not affected by other risk factors. These findings showed anemia was indeed an independent risk factor for rapid eGFR decline in type 2 diabetic patients. A recent multicenter cohort study in Japan revealed that circulating Hb concentration, associated with tubulointerstitial injury, could be a predictor for DKD progression (37), which was consistent with our results.

This study has a number of limitations. First, blood pressure, as an important element in the patients of DKD, was not included in the analysis. Second, this is a retrospective study with a relatively short follow-up. Third, numbers of patients were limited in the study, especially those had severe anemia and lower eGFR. Large scale prospective study is still needed to evaluate the value of anemia in predicting the progression of DKD.

## 5 Conclusion

Taken together, anemia is common in patients with type 2 diabetes, which might be a predictor for rapid eGFR decline. In clinical management, anemia should be paid more attention in diabetic patients. Specifically, closely monitoring of renal function may be necessary for diabetic patients with anemia.

## Data availability statement

The raw data supporting the conclusions of this article will be made available by the authors, without undue reservation.

## Ethics statement

The studies involving human participants were reviewed and approved by Ethics Committee at the Huashan Hospital of Fudan University. The patients/participants provided their written informed consent to participate in this study.

## Author contributions

MH and JW designed the study. LX and XS wrote the manuscript. YYu, XW and JW collected the data. YYang, XH, MW and FS analyzed the data. WG, MW, WL, HW, ZZ and YL gave many advices in the study design. All authors contributed to the article and approved the submitted version.
